# Lack of association between four SNPs in the *SLC22A3-LPAL2-LPA* gene cluster and coronary artery disease in a Chinese Han population: a case control study

**DOI:** 10.1186/1476-511X-11-128

**Published:** 2012-10-04

**Authors:** Xiaofei Lv, Yuan Zhang, Shaoqi Rao, Fengqiong Liu, Xiaoyu Zuo, Dongfang Su, Min Wang, Min Xia, Honghui Guo, Dan Feng, Changjiang Hong, Dan Li, Wenjun Ma, Ping Ouyang, Xinrui Li, Xiang Feng, Yan Yang, Wenhua Ling, Jian Qiu

**Affiliations:** 1 Guangdong Provincial Key Laboratory of Food, Nutrition and Health, Department of Nutrition, School of Public Health, Sun Yat-Sen University (Northern Campus), 74 Zhongshan Road 2, Guangzhou, 510080, China; 2 Department of Cardiology, Guangzhou Region General Hospital, 111 Liuhua Road, Guangzhou, 510070, China; 3 Institute of Medical Systems Biology; Department of Medical Statistics and Epidemiology, School of Public Health, Guangdong Medical College, Dongguan, Guangdong, 523808, China; 4 Department of Medical Statistics and Epidemiology, School of Public Health, Sun Yat-Sen University (Northern Campus), 74 Zhongshan Road 2, Guangzhou, 510080, China; 5 Department of Food Science, Yingdong College of Bioengineering, Shaoguan University, Daxue Road, Shaoguan, 512005, Guangdong Province, China; 6 Department of Nutrition, Guangdong Provincial People’s Hospital, 106 Zhongshan Road 2, Guangzhou, 510080, China

**Keywords:** Association study, CAD, Lp(a), *SLC22A3-LPAL2-LPA*, SNP

## Abstract

**Background:**

Lipoprotein (a) (Lp [a]) is known being correlated with coronary artery disease (CAD). The *SLC22A3-LPAL2-LPA* gene cluster, relating with modulating the level of plasma Lp (a), has recently been reported to be associated with CAD in Caucasians. The purpose of this study was to verify whether this finding can be expanded to the Chinese Han population.

**Methods and Results:**

Using a Chinese Han sample, which consisted of 1012 well-characterized CAD patients and 889 healthy controls, we tested the associations of four SNPs (rs2048327, rs3127599, rs7767084 and rs10755578) in the *SLC22A3-LPAL2-LPA* gene cluster, and their inferred haplotypes with the risk of CAD. Allelic, genotypic and haplotype association analyses all showed that the gene cluster was not associated with CAD in this Chinese Han sample.

**Conclusions:**

We for the first time explored the association of the four SNPs in the *SLC22A3-LPAL2-LPA* gene cluster with CAD in a large Chinese Han sample. Nevertheless, this study did not reveal any significant evidence of this gene cluster to increase the risk of CAD in this population.

## Background

Coronary artery disease (CAD) is now widespread and becoming a heavy burden for both developed and developing countries. A long list of susceptibility loci for CAD has been identified in previous genetic studies. Most recently, the gene cluster aligned by *solute carrier family 22 member 3 (SLC22A3), lipoprotein(a)-like 2 (LPAL2),* and *lipoprotein(a) (LPA)*, known as *SLC22A3-LPAL2-LPA* on chromosome 6q26-27, attracted much attention, possibly due to its capability to regulate the plasma level of lipoprotein(a) (Lp(a)) [1].

Lp(a), a low-density lipoprotein (LDL)-like particle synthesized in the liver [2], has been well known as an independent risk factor for CAD [3-6]. Its protein component-apolipoprotein(a)[apo(a)], accounts for 91% variation of the plasma Lp(a) concentration [7]. *LPA*, one member of the *SLC22A3-LPAL2-LPA* gene cluster*,* encodes apo(a) [5], and is associated with Lp(a) levels, explaining up to 36% of Lp(a) variance in European-descent [5,8,9]. In addition, the hapoltype formed by four SNPs (rs2048327, rs3127599, rs7767084 and rs10755578) in this region is also related to the plasma level of Lp(a) [1].

Researchers tried to explore the association between the *SLC22A3-LPAL2-LPA* gene cluster and CAD. Tregouet *et al.* identified this region as a risk cluster for CAD in the genome-wide haplotype study (GWHS) in six white populations [1]. Koch *et al.* demonstrated that the gene cluster was a strong susceptive locus for MI in the European [10]. However, the research of Qi *et al.* did not confirm the association between haplotypes in the *SLC22A3-LPAL2-LPA* region and nonfatal acute MI risk in Hispanics [11].

These contrary data indicate that more large-scale and independent studies should be performed to confirm the association between this cluster and CAD and verify whether this finding can be expanded to other populations. Up to date, there is no report on the correlation between *SLC22A3-LPAL2-LPA* and CAD in Chinese population, which takes up one fifth of the human population.

## Results

### Power analysis

We performed a statistical power analysis using the PS program to verify whether the recruited samples could provide adequate power in identifying the association between the SNPs and CAD. Under the assumption of odd ratio being 1.236, and the risk allele frequency being 0.341, as previously reported [1], our sample size with 1012 well-characterized CAD cases and 889 healthy controls can provide a statistical power of 88.0% and 71.2% at the nominal type I error rate of 0.05 and 0.01 respectively. The power analysis indicated that our sample size is sufficient for identifying the modest-effect-size SNP.

### Characteristics of participants

This case–control study included 1012 CAD patients and 889 healthy controls. Characteristics of the participants are summarized in Table 1. Compared with controls, the CAD patients were older, more likely to have a higher BMI, and to be current smokers.

**Table 1:** Characteristics of the participants

**Characteristics**	**CHD cases**	**Healthy controls**	***P***
Sample Size	1012	889	*-*
Male (%)	68.1% (689/1012)	63.9 (568/889)	*0.054*
Age	63.74±11.13	59.79±5.63	*<0.001*
Current Smokers (%)	63.75±5.71	59.92±5.62	*<0.001*
BMI	23.84±3.27	23.29±3.11	*<0.001*

Data are shown as mean ± SD or percentage.

Continuous data were tested using 2-tailed Student *t*-test and categorical data were tested using a Chi-square test (with df = 1) for difference between CHD cases and healthy controls.

### Characteristics of four SNPs

**Figure 1:**
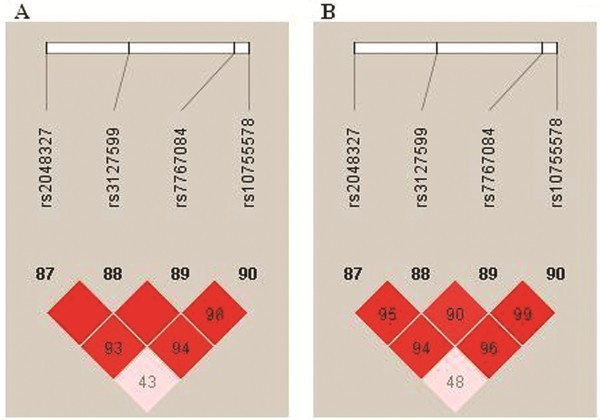
**LD plots of the four SNPs in the *SLC22A3-LPAL2-LPA *region. **LD patterns between four SNPs, rs2048327, rs3127599, rs7767084 and rs10755578, were derived from the genotyping data from the Han Chinese CHD patients (**A**) and healthy control (**B**), respectively. The pairwise correlation between the SNPs was measured as D’ and shown (×100) in each diamond.

All the call rates for the 4 SNPs were above 99%. All the four SNPs tested were polymorphic, with minor allele frequency ranging from 0.124 to 0.412, and in agreement with Hardy-Weinberg equilibrium. The linkage disequilibrium between rs2048327 and rs10755578 was relatively low (D’= 0.43 in cases, and D’= 0.48 in controls). D’ values between other SNPs ranged from 0.94 to 1. The information about the four SNPs was shown in Table 2 and Figure 1.

**Table 2:** **Information about the four SNPs in the *SLC22A3-LPAL2-LPA *region**

**SNP**	**Chromosome**	**Gene**	**Minor allele**	**MAF**	*P*-HW*	**Call rate**
rs2048327	6q26-q27	SLC22A3	C	0.410	0.78	99.2%
rs3127599	6q26-q27	LPAL2	T	0.124	0.52	99.7%
rs7767084	6q26-q27	LPA	C	0.261	0.22	99.7%
rs10755578	6q26-q27	LPA	C	0.412	0.36	99.8%

MAF, minor allele frequency.

*P*-HW*, *P *value for Hardy-Weinberg disequilibrium analysis in controls.

### Allelic association and genotypic association

First, the associations between polymorphisms of rs2048327, rs3127599, rs7767084, rs10755578 and CAD were evaluated. OR and 95% CI for each SNP were calculated with the ancestral allele as the reference, and the statistical significance was defined by the permutation test mentioned in the method section. There was no significant association between any allele of the four SNPs and CAD (*P-obs* = 0.23-0.50, OR: 0.92-1.10). Permutation *P* values calculated using 100 000 Monte Carlo simulations were of no significance, either (as shown in Table 3).

**Table 3:** **Allelic association of four SNPs in *SLC22A3-LPAL2-LPA *with CHD in a Han Chinese population**

	**Frequency**		**Allele/Risk**	**OR**	***†P***	***‡P***
**SNP**	**Case**	**Control**	**Ancestral**	**(95%CI)**	***-obs***	**-emp**
rs2048327	0.405	0.417	C/T	0.95(0.84-1.08)	0.45	0.84
rs3127599	0.129	0.118	T/C	1.10(0.91-1.34)	0.33	0.69
rs7767084	0.265	0.255	C/T	1.05(0.91-1.22)	0.5	0.87
rs10755578	0.579	0.599	G/C	0.92(0.81-1.05)	0.23	0.53

Ancestral allele was retrieved from dbSNP database.

*†P-obs* , uncorrected *P* value. *‡P-emp* , permutation *P* value calculated using 100 000 Monte Carlo simulations.

OR, odds ratio; CI, confidence interval.

We further examined the association between individual SNP and CAD risk under additive, dominant or recessive model, respectively. In the univariate logistic regression, none of the four SNPs showed significant association with CAD risk (*P* = 0.09-0.84), and further adjustment for the conventional risk factors such as age, sex, smoking and BMI did not change the results (*P* = 0.12-0.77). The results were list in Table 4.

**Table 4:** **Assessment of association between four SNPs in *SLC22A3-LPAL2-LPA *region with CHD**

		**Without Adjustment†**		**With Adjustment ‡**	
**SNP**	**Model**	***P***	**OR (95%CI)**	***P***	**OR (95%CI)**
rs2048327	Additive	0.44	0.95(0.83-1.08)	0.56	0.96(0.84-1.10)
	Dominant	0.84	1.02(0.94-1.24)	0.70	1.04(0.85-1.27)
	Recessive	0.09	0.81(0.63-1.03)	0.12	0.82(0.63-1.05)
rs3127599	Additive	0.32	1.10(0.91-1.34)	0.53	1.07(0.87-1.31)
	Dominant	0.37	1.10(0.89-1.36)	0.59	1.60(0.85-1.33)
	Recessive	0.50	1.32(0.59-2.96)	0.59	1.26(0.55-2.92)
rs7767084	Additive	0.50	1.05(0.91-1.22)	0.19	1.13(0.94-1.37)
	Dominant	0.26	1.11(0.93-1.33)	0.19	1.13(0.94-1.37)
	Recessive	0.60	0.91(0.64-1.29)	0.60	0.91(0.63-1.31)
rs10755578	Additive	0.21	0.92(0.80-1.05)	0.29	0.93(0.81-1.07)
	Dominant	0.84	0.98(0.76-1.25)	0.77	0.96(0.74-1.25)
	Recessive	0.10	0.85(0.70-1.03)	0.20	0.88(0.72-1.07)

†*P* values were obtained from univariate logistic regression model where the SNP predictor was coded according to three different genetic models.

‡*P* values were multivariate obtained from logistic regression model after adjustment for gender, age, smoking and BMI.

OR, odds ratio; CI, confidence interval.

### Haplotype association analysis

The frequencies of haplotypes from different blocks were estimated and all the haplotypes with a frequency above 1% were included in the following analysis. In the haplotype association analysis, one haplotype was treated as a single variant, and all the other haplotypes were collapsed into the alternative allele to test its association with CAD. Age, sex, smoking and BMI were adjusted. The results were shown in Table 5.

**Table 5:** Assessment of association between haplotypes made up of four SNPs with CHD

		**Frequency**				
**SNPs**	**Haplotype**	**Case**	**Control**	**OR**	**†*P*-obs**	**‡*P*cpemp**
rs2048327|rs3127599					0.14*	0.36*
|rs7767084|rs10755578	CCCC	0.255	0.246	1.06	0.47	0.99
	TTTC	0.123	0.109	1.12	0.30	0.95
	CCTC	0.025	0.033	0.71	0.11	0.64
	TCTC	0.013	0.008	1.75	0.16	0.78
	CCTG	0.128	0.14	0.92	0.40	0.98
	TCTG	0.457	0.465	0.96	0.61	1
rs2048327|rs3127599					0.28*	0.60*
|rs7767084	CCC	0.258	0.25	1.05	0.52	1
	TTT	0.129	0.116	1.09	0.40	0.98
	CCT	0.15	0.17	0.88	0.15	0.76
	TCT	0.463	0.464	0.99	0.91	1
rs2048327|rs3127599					0.17*	0.41*
|rs10755578	TTC	0.125	0.114	1.09	0.43	1
	CCC	0.274	0.274	1.01	0.95	1
	TCC	0.023	0.014	1.82	0.04	0.33
	CCG	0.13	0.143	0.92	0.38	0.98
	TCG	0.447	0.455	0.97	0.62	1
rs2048327|rs7767084					0.18*	0.43*
|rs10755578	CCC	0.257	0.249	1.05	0.56	1
	CTC	0.025	0.032	0.68	0.11	0.66
	TTC	0.134	0.117	1.15	0.20	0.85
	CTG	0.126	0.14	0.90	0.32	0.96
	TTG	0.458	0.463	0.97	0.69	1
rs3127599|rs7767084					0.43*	0.78*
|rs10755578	CCC	0.262	0.251	1.07	0.37	0.98
	TTC	0.122	0.108	1.11	0.33	0.96
	CTC	0.038	0.04	0.90	0.56	1
	CTG	0.579	0.601	0.92	0.27	0.93
rs2048327|rs3127599					0.66*	0.93*
	TT	0.127	0.117	1.07	0.52	1
	CC	0.405	0.416	0.97	0.62	1
	TC	0.468	0.466	1.01	0.89	1
rs3127599|rs7767084					0.35*	0.69*
	CC	0.265	0.254	1.07	0.37	0.98
	TT	0.128	0.117	1.07	0.50	1
	CT	0.607	0.629	0.92	0.22	0.88
rs7767084|rs10755578					0.37*	0.71*
	CC	0.264	0.254	1.06	0.42	1
	TC	0.158	0.147	1.05	0.62	1
	TG	0.579	0.599	0.92	0.24	0.91
rs2048327|rs7767084					0.24*	0.53*
	CC	0.257	0.248	1.06	0.49	1
	CT	0.152	0.172	0.87	0.14	0.73
	TT	0.591	0.581	1.03	0.70	1
rs2048327|rs10755578					0.16*	0.39*
	CC	0.274	0.279	0.97	0.67	1.
	TC	0.146	0.122	1.27	0.03	0.29
	CG	0.130	0.138	0.96	0.72	1
	TG	0.450	0.461	0.94	0.41	0.98
rs3127599|rs10755578					0.39*	0.73*
	TC	0.125	0.115	1.06	0.57	1
	CC	0.298	0.288	1.05	0.53	1
	CG	0.578	0.597	0.93	0.30	0.95

†*P-obs*, uncorrected *P* value. *‡P-emp*, permutation *P* value calculated using 100 000.

Monte Carlo simulations.

* Uncorrected *P* value and permutation *P* value for a single omnibus test, which jointly estimate a testing all haplotype effects at the position, df = H-1, if there are H haplotypes at the position.

OR, odds ratio. OR for every haplotype was calculated taking all the rest haplotypes together as reference.

Only two of all the tested haplotypes, TCC (which was made up by rs2048327, rs3127599 and rs10755578), and TC (which was made up by rs2048327 and rs10755578), reached the single-point significance level (*P-obs* = 0.04 and 0.03). However, the two findings could not survive over multiple test corrections. The *P*-values for the two haplotypes after the permutation test using 100 000 Monte Carlo simulations were 0.33 and 0.29, respectively. Moreover, the overall tests for all haplotypes formed by the three and the two SNPs did not achieve significance (*P-obs* = 0.17, with df = 4 and *P-obs* = 0.16, with df = 3), either. Haplotypes TC and TG formed by rs2048327 and rs10755578 were reported to be the risk haplotypes in a Japanese sample [12]. However, haplotype TG showed no significant association with CAD (*P-obs* = 0.41, *P-emp* = 0.98) in this Chinese Han sample, as well as haplotype TC mentioned above (as shown in Table 5).

In previous studies of European populations, the most common haplotype formed by the four SNPs was TCTC, and haplotypes CCTC, CTTG and TTTC were found to be associated with increased risk of CAD [1,10]. In this study, the most common haplotype was TCTG and the haplotype CTTG was not found. Haplotypes CCTC and TTTC showed no significant association with CAD (*P-obs* = 0.11, *P-emp* = 0.64 and *P-obs* = 0.30, *P-emp* = 0.95, separately). With the most common haplotype TCTG as the reference, OR and 95% CI for haplotypes CCTC and TTTC were 0.77 (0.45-1.32) and 1.15 (0.86-1.53). Furthermore, the corresponding OR and 95% CI for haplotypes CCTC and TTTC were 0.47 (0.17-1.30) and 0.694 (0.28-1.74), when the haplotype TCTC was used as the reference, which could not reach statistical significance, either. Data were not shown here.

## Discussion

In 2009, Tregouet *et al.* identified the *SLC22A3-LPAL2-LPA* gene cluster as a risk cluster and haplotypes CTTG and CCTC formed by rs2048327, rs3127599, rs7767084 and rs10755578 as risk haplotypes for CAD in six White populations [1]. From then on, several GWHS have focused on this hot spot. In a study consisted of 3657 patients with MI and 1211 control individuals, Koch *et al.* observed significant association between haplotypes formed by the same four SNPs in the *SLC22A3-LPAL2-LPA* region and MI (P = 0.0005), and found 3 risk haplotypes (CTTG, CCTC, and TTTC) [10]. Later, Sawabe M etal analyzed rs2048327 (C/T) and rs10755578 (C/G) in 1,150 Japanese autopsy cases, and ascertained that haplotypes TC and TG worked as risk factors for both coronary sclerosis and CAD [12]. In addition, Shaw *et al.* found that genetic variants at the *SLC22A3-LPAL2-LPA* locus were associated with decreased early-outgrowth colony-forming units, thereby increased the risk of MI [13], which may support the findings in population studies mentioned above. However, Qi *et al.* did not confirm the association of haplotypes at the *SLC22A3-LPAL2-LPA* locus with nonfatal MI risk in Hispanics [11].

For gene association studies, repeating previous findings across different populations is essential for exploring the full scape of their pathogenic nature. To date, there is no study focusing on the association between CAD and the *SLC22A3-LPAL2-LPA* gene cluster in Chinese people. Our study for the first time attempted to explore such association in Chinese Hans. We evaluated the association between four SNPs in this gene cluster and CAD by examming all kinds of associations (allelic, genotypic and haplotype). Nevertheless, we did not identify any significant evidence to link this gene cluster to CAD risk in this Chinese Han sample. The genotypic and allelic association between individual SNP and CAD drawn from our data were consistent with results from previous GWHS [1]. Whereas, there are differences exist between our study and previous studies. The most common haplotype we found was TCTG instead of TCTC, which was reported in European populations [1,10,11]. Moreover, we did not confirm the association of haplotypes CTTG, CCTC, and TTTC with CAD reported in European populations [1,10]. In addition, we did not find any association between CAD and two haplotypes TC and TG composed by rs2048327 and rs10755578, which was inconsistent with the results from a Japanese study [12].

There are many reasons for heterogeneity in genetic association studies. Ethnic differences in genetic structure may produce different LD, thereby differences in the significance of the association test, which also exist in other genetic association studies [14]. Besides, differences in environmental, dietary or behavioral factors may also partially explain the heterogeneity in the genetic associations across ethnicities [15,16]. Furthermore, different disease definitions under different criteria may also be partly responsible for the variation between studies.

To limit the potential influence of factors mentioned above, we carefully designed and implemented this study. First, we used an adequate sample with enough statistical power, to detect the genetic association, therefore, the discrepancies between our study and others in different populations may be more likely due to the ethnic differences in genetic structure. Second, we performed multivariate logistic model to adjust several possible covariates, such as age, gender, smoking status and BMI. Moreover, we identified the case subjects in a strict accordance with a generally accepted definition of CAD and excluded patients taking niacin which could decrease the plasma level of Lp(a) and/or patients with diabetes since diabetes status was reported to attenuate the relation between Lp(a) and cardiovascular risk [17].

Despite our study was well organized, several limitations still exist in this exploratory study. First, as a complex disease, many factors may contribute to CAD, such as environmental and polygenic backgrounds, dietary and behavioral factors, hence, the genetic parameter estimates (odd ratios, risk allelic or genotype frequencies) may be biased. In addition, uncontrolled confounding factors may lead to spurious associations. Although many important confounding factors were controlled or adjusted in our analysis, some potential confounders, such as lipid level, were unavailable for a large number of subjects and thus not controlled.

## Conclusions

Our study for the first time explored the association between CAD and the four SNPs in the *SLC22A3-LPAL2-LPA* gene cluster in Chinese Hans. We found no allelic, genotypic and haplotype association between the four SNPs in the *SLC22A3-LPAL2-LPA* gene cluster and CAD.

## Methods

### Subjects

The method we recruited participants was described prevously [18]. Briefly, all subjects were of the ethnic Han origin and are not related to each other. Cases and controls are gender-frequency matched. Patients with CAD were recruited from the Guangzhou Military Region General Hospital and the healthy controls were randomly selected from several communities in Guangzhou. CAD was diagnosed if any of the following criteria was met: (1) confirmed MI; (2) ≥50% stenosis in at least one coronary vessel at angiography; (3) a validated history of percutaneous transluminal coronary angioplasty or coronary artery bypass graft surgery and pectoris; (4) primarily diagnosed by symptoms and later confirmed by at least one non-invasive provocation test, e.g. scintigraphy or treadmill test [19]. Patients with diabetes and/or those taking niacin were excluded. Full hospital records were reviewed to confirm the diagnosis. At the enrolment, anthropometric measures and drug uses for both cases and controls were collected by well-trained interns and physicians. Body mass index (BMI) was calculated using the formula: BMI=weight in kilograms/ (height in meters×height in meters). The study was approved by the Ethics Committees of the participating hospitals and institutions. All participants have signed the written informed consent forms. The investigation conformed to the principles outlined in the Declaration of Helsinki.

### SNP Genotyping

Genomic DNA was isolated from peripheral blood lymphocytes using the TIANamp Blood DNA Kit (Tiangen Inc; Beijing; China). The four SNPs rs204832, rs3127599, rs7767084 and rs10755578 were genotyped using Illumina Golden Gate Genotyping Bead Chips (Illumina Inc; San Diego; USA) (http://www.Illumina.Com/), which uses illumiCodes, unique 23-bp single stranded DNA oligos, to correctly identify each DNA as well as the loci being interrogated [20]. First, prepared DNA samples were amplified using universal PCR primers labeled with Cy3 and Cy5 fluorescent dyes, and the resulting fluorescently labeled PCR product was then hybridized to a Universal BeadChip, which contained randomly assembled universal beads, each displaying an illumiCode corresponding to specific loci.

The quality for SNP genotyping was assured by independently replicating the genotyping and allelic calls of 30 randomly selected samples. The results from quality control were in perfect agreement with the initial genotyping results. In addition, all the DNA samples for cases and controls were run in the same batches.

### Statistical analysis

Continuous covariates were expressed as meaxn ± SD, and the differences between cases and controls were analyzed by independent *t*-test or Mann–Whitney *U* test. Categorical variables were summarized as frequency (percentage) and analyzed by Chi-square test. Genotype coding method of Lewis was used to build additive, dominant and recessive genetic models [21]. Odds ratio (OR) and Wald 95% confidence interval (CI) were calculated using the homozygote of ancestral allele as reference. The ancestral alleles were defined by the dbSNP database (http://www.ncbi.nlm.nih.gov/snp). Genotypic association between each SNP and CAD was analyzed by using univariate (with only SNP included) and multivariate BMI logistic regression under additive, dominant and recessive genetic models. In the multivariate analysis, age, gender, smoking and BMI were also modeled and adjusted. All statistical analyses mentioned above were performed with the SPSS 13.0 (SPSS Inc; Chicago; USA).

Prior to the experiment, the case–control design and the sample size were evaluated by the PS program, a software for power analysis [22]. The Hardy-Weinberg equilibrium test and linkage disequilibrium (LD) between the SNPs were analyzed by employing Haploview 4.2 (http://www.broad.mit.edu/mpg/haploview). The extent of disequilibrium was expressed by D’. Haplotype frequency estimation, allelic and haplotype association analyses were implemented and then followed by permutation analysis with 100 000 Monte Carlo simulations by using PLINK software (http://pngu.mgh.harvard.edu/~purcell/plink). In the allelic association analysis, OR and 95%CI were calculated using the ancestral allele as reference. The associations between all the haplotypes drawn from every two, every three or all four SNPs and CAD were analyzed, except for those with frequency below 1%.

## Abbreviations

Lp(a): Lipoprotein(a); CAD: Coronary artery disease; SLC22A3: Solute carrier family 22 member 3; LPAL2: Lipoprotein(a)-like 2; LPA: Lipoprotein(a); Lp(a): Lipoprotein(a); MI: Myocardial infarction; apo(a): Apolipoprotein(a); GWHS: Genome-wide haplotype study; BMI: Body mass index; OR: Odds ratio; CI: Confidence interval; LD: Linkage disequilibrium.

## Competing interests

The author’s declare that they have no competing interests'.

## Authors’ contributions

JQ, SR, WL and MX conceived of the project and participated in its design, revised the paper, and they gave final approval of the version to be published. YZ, XL, MW, DS and XL collected clinical data and extracted DNA. XL and JQ carried out statistical analysis and wrote the manuscript. FL and XF took charge of genotyping, and helped to draft the manuscript. XZ, DF, HG, DL,Y Y and PO helped to statistical analysis and revised the paper. XL, YZ and SR contributed equally to this work. All authors read and approved the final manuscript.
